# Host in reserve: The role of common shrews (*Sorex araneus*) as a supplementary source of tick hosts in small mammal communities influenced by rodent population cycles

**DOI:** 10.1002/ece3.8776

**Published:** 2022-04-11

**Authors:** Nicolas De Pelsmaeker, Lars Korslund, Øyvind Steifetten

**Affiliations:** ^1^ Department of Nature, Health and Environment University of Southeastern Norway Bø Norway; ^2^ Department of Natural Sciences University of Agder Kristiansand Norway

**Keywords:** bank vole, common shrew, *Ixodes ricinus*, *Ixodes trianguliceps*, *Myodes glareolus*, *Sorex araneus*, ticks

## Abstract

Rodents often act as important hosts for ticks and as pathogen reservoirs. At northern latitudes, rodents often undergo multi‐annual population cycles, and the periodic absence of certain hosts may inhibit the survival and recruitment of ticks. We investigated the potential role of common shrews (*Sorex araneus*) to serve as a supplementary host source to immature life stages (larvae and nymphs) of a generalist tick *Ixodes ricinus* and a small mammal specialist tick *I*. *trianguliceps*, during decreasing abundances of bank voles (*Myodes glareolus*). We used generalized mixed models to test whether ticks would have a propensity to parasitize a certain host species dependent on host population size and host population composition across two high‐latitude gradients in southern Norway, by comparing tick burdens on trapped animals. Host population size was defined as the total number of captured animals and host population composition as the proportion of voles to shrews. We found that a larger proportion of voles in the host population favored the parasitism of voles by *I*. *ricinus* larvae (estimate = −1.923, *p* = .039) but not by nymphs (estimate = −0.307, *p* = .772). *I*. *trianguliceps* larvae did not show a lower propensity to parasitize voles, regardless of host population composition (estimate = 0.875, *p* = .180), while nymphs parasitized shrews significantly more as vole abundance increased (estimate = 2.106, *p* = .002). These results indicate that common shrews may have the potential to act as a replacement host during periods of low rodent availability, but long‐term observations encompassing complete rodent cycles may determine whether shrews are able to maintain tick range expansion despite low rodent availability.

## Graphical abstract

Climate change is causing ticks to expand their distribution range both northwards and upwards in altitude. This study investigates whether common shrews (*Sorex araneus*) can serve as supplementary hosts for ticks in regions characterized by large‐amplitude rodent population cycles. The results indicate that ticks may have the potential to continue their range expansion when shrews are present in periods when rodent abundance is low.

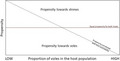

## 1 INTRODUCTION

The effects of climate change have played a part in allowing several parasites to expand their range toward new areas (Kutz et al., 2013; Randolph, 2009; Sonenshine, 2018; Välimäki et al., 2010), and in recent decades, ticks (Acari: *Ixodidae*) have been observed to have shifted their distribution limit both northwards (Jore et al., 2011, 2014; Lindgren & Gustafson, 2001; Lindgren et al., 2000; Ogden et al., 2014) and upwards in altitude (Daniel et al., 2003; Jore et al., 2011; Martello et al., 2014; Materna et al., 2008; Mejlon, 2000). Ticks are vectors for a number of pathogens capable of threatening the health of both humans (Cotté et al., 2008; Grzeszczuk et al., 2004; Liebisch et al., 1998; Paul et al., 1987; Pusterla et al., 1999; Süss et al., 2006) and animals (Cotté et al., 2008; Donnelly & Peirce, 1975; Pusterla et al., 1999), and an expansion in the distribution range of ticks has the potential to introduce new tick‐borne pathogens into previously unaffected areas and regions, as well as increase the prevalence of existing pathogens. The current expansion seen among tick species is likely to continue in the future. With higher expected temperatures, ticks might survive in new areas where environmental conditions previously unsuitable to their establishment may become increasingly favorable (Ogden et al., 2006, 2014). Aside from being dependent on hospitable climatic conditions, ticks, as obligate parasites, also depend on the availability of adequate hosts to feed and reproduce. Yet, questions regarding how host availability can affect the abundance of ticks near their range limit still remain, and the role of hosts therein is rarely considered when habitat suitability for ticks is evaluated (Estrada‐Peña & de la Fuente, 2017).

Depending on their life stage, ticks may feed on different hosts, and immature tick stages (larvae and nymphs) often feed on small mammals (Nilsson & Lundqvist, 1978; Paziewska et al., 2010; Shaw et al., 2003). Rodents play an important role in maintaining tick populations, as well as perpetuating the infection cycle among ticks, pathogens, and wild reservoirs (Ambrasiene et al., 2009; Bown et al., 2006; Boyard et al., 2008; Brunner & Ostfeld, 2008; Estrada‐Pena et al., 2005). In the northern parts of Europe, rodents typically undergo multi‐annual population cycles (Hörnfeldt et al., 2006), and the amplitude of these cycles is more pronounced at higher latitudes and altitudes (Andreassen et al., 2020; Bjørnstad et al., 1995). During the low phase of the cycle, some species can reach very low population levels (Boonstra et al., 1998), and such recurring periods of low small rodent host availability could potentially limit the presence of ticks due to reduced survival and recruitment.

The sheep tick *Ixodes ricinus* is the most common tick species in Europe (Petney et al., 2012) and the most important vector for tick‐borne infections in humans (Estrada‐Peña & Jongejan, 1999). *I*. *ricinus* is an exophilic species, searching for a host in open vegetation (Gern et al., 2008). Considered a generalist, it feeds on a wide variety of hosts (Hillyard, 1996; Medlock et al., 2013), but the bank vole (*Myodes glareolus*), which is commonly found in most of Europe (Banach, 1988; Haapakoski & Ylönen, 2010; Mazurkiewicz, 1994; Stenseth, 1985), is one of the most important host species to immature stages of *I*. *ricinus* (Andersson et al., 2014; Mysterud et al., 2015; Tälleklint et al., 1993). Bank vole population cycles are typically 3 to 4 years long (Hörnfeldt, 1978; Kaikusalo, 1972), and studies have previously found that bank vole dynamics can influence the prevalence of a zoonotic disease (Milhano et al., 2017). Low phases in bank vole cycles could possibly pose a challenge to ticks due to a deficit in available hosts, which might eliminate the potential for viable tick populations, and thus their ability to successfully spread to new areas. However, no tick species is solely dependent on small rodents as hosts, and the presence of other hosts could potentially mitigate any negative effects of low rodent numbers. Another widespread and commonly parasitized small mammal in northern Europe is the common shrew (*Sorex araneus*) (Bown et al., 2011; Mysterud et al., 2015). Common shrews occupy the same habitats as bank voles (Churchfield, 1990), and are parasitized by similar tick fauna (Arthur, 1963; Randolph, 1975a). Both host species have a similar pathogen reservoir potential and contribute to the maintenance of the infection cycle among pathogens, ticks, and their hosts (Bakhvalova et al., 2001; Bown et al., 2011; Gern et al., 1998; Kozuch et al., 1967). Common shrew populations fluctuate erratically, but do not seem to undergo periodic cycles, and the fluctuations appear to be less pronounced than what is the case for rodents (Buckner, 1969; Henttonen et al., 1989). During the low phase in rodent cycles, generalist ticks might therefore parasitize the relatively more abundant shrews, thus being less vulnerable to low rodent densities where strong population fluctuations occur.

Tick species specializing on small mammals, such as *Ixodes trianguliceps*, parasitize small mammals during all life stages (Hillyard, 1996), and may therefore be even more influenced by large fluctuations of small rodent hosts. *I*. *trianguliceps* is considered a nidiculous (endophilic) tick, spending its off‐host time searching for a host within the burrows of small mammals (Hillyard, 1996; Randolph, 1975a). This difference in ecology, compared to generalist (exophilic) ticks, further reduces the chances of encounters with other potential hosts. With a reduction in rodent availability, the specialist *I*. *trianguliceps*, feeding only on small mammals, would be even more reliant on the availability of other small mammal species for survival, whereas the generalist *I*. *ricinus* may parasitize alternative host species such as larger mammals or birds.

**FIGURE 1:**
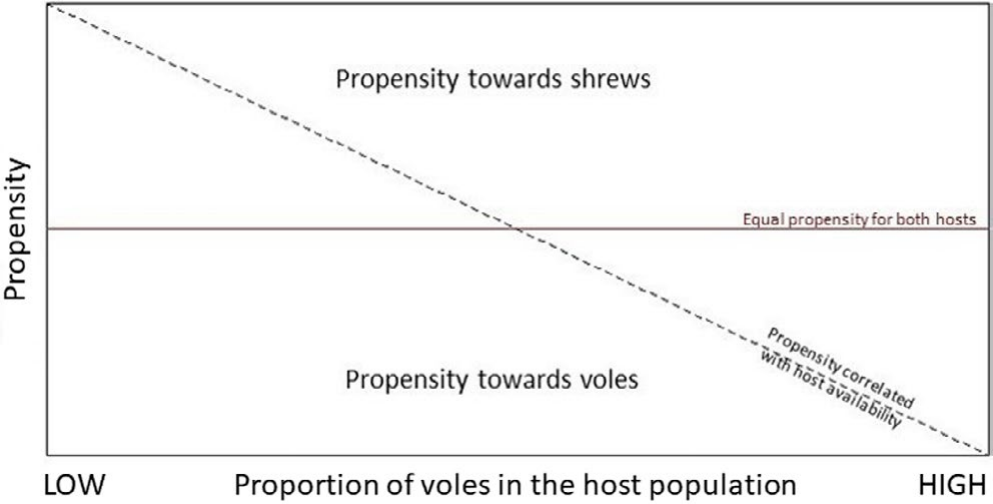
Conceptual diagram of the relationship between propensity of parasitism and host population composition. Positive values represent a propensity toward shrews, negative values a propensity toward voles. The horizontal red line indicates no difference in propensity toward either host type, regardless of host population composition. The dashed diagonal line indicates a propensity disproportional to the population composition

The effect of host population dynamics and its relation to tick expansion is presently an underexplored area of research, but such knowledge could prove important when predicting future expansion of ticks, and therein the potential risk for disease transmission. Using bank voles and common shrews as model species, we investigate whether host utilization of generalist and small mammal specialist ticks are influenced by host availability in regions near their distribution limit, and where rodent population cycles are characterized by large fluctuations in population size. The goal was to assess whether voles are the preferred host and if shrews have the potential to act as an alternative host in periods of low rodent abundance. We tested whether ticks were strictly opportunistic, parasitizing hosts purely based on their relative abundance, or whether a propensity toward a certain host type existed, that is, if we see a pattern similar to a predator–prey functional response (Holling, 1959; Solomon, 1949), and if any propensity was dependent on overall small mammal abundance. This may differ between tick species and life stages. We hypothesize that opportunistic ticks would have an equal chance of parasitizing a passing host, regardless of the host species, and therefore no pattern in mean burden ratios (propensity) is to be expected (Figure 1). A decline in the proportion of voles in the host population is expected to result in higher mean burdens on that host type.

## 2 METHODS

Two mountain slopes in the southeastern and western part of Norway were selected for host trapping, spanning three seasons (spring, summer, and autumn) over 2 consecutive years (2017 and 2018). The southeastern area of Lifjell (N59°26.495′ E9°0.603′) is a southern facing mountain slope characterized by a continental climate located within the boreonemoral to southern boreal zone, dominated by mostly homogenous mixed forests. Birch (*Betula pubescens*) and spruce (*Picea abies*) dominate below the tree line, which is situated between 800 and 900 m.a.s.l. (meters above sea level), and blueberry (*Vaccinium myrtillus*) is the dominant species at ground layer. The vegetation above the tree line consists primarily of common heather (*Calluna vulgaris*). The western study area is a northern facing mountain slope located in the Erdal valley near Lærdalsøyri (N61°05.817′ E7°24.688′, hereafter referred to as Lærdal). Characterized by a more maritime climate, this area typically has milder winters and cooler summers. The tree line is situated between 900 and 1000 m.a.s.l., and below the tree line homogeneous deciduous forests dominate, consisting primarily of birch and alder (*Alnus glutinosa*). Different tall perennial herbs, ferns, and blueberries constitute the ground layer. Above the treeline, common heater, dwarf birch (*Betula nana*), common juniper (*Juniperus communis*), and crowbery (*Empetrum nigrum*) dominate.

On both mountain slopes, 10 capturing stations were set up, ranging from 100 to 1000 m.a.s.l. At every station, two trapping grids consisting of 20 traps each were deployed. The grids were located a minimum of 100 m apart. The traps in each grid were arranged in 4 by 5 rows, with 10 m spacing in between each trap. One plot was equipped with live traps (Ugglan Special Nr. 2, Grahnab AB, Sweden, www.grahnab.se) and baited with slices of apple and whole oats. A layer of sawdust was provided as insulation on the trap floor. The other plot was equipped with lethal traps (Rapp2 Mousetrap, www.rappfellene.no), and baited with peanut butter. A previous study has shown that both trap types perform similarily in terms of trapping efficiency and tick burdens (De Pelsmaeker et al., 2020). Humidity and temperature were recorded at station level every hour for the duration of the trapping period, using a TinyTag Plus 2 – TGP 4017 datalogger, housed in a DataMate instrument cover ACS‐5050, mounted approximately 50 cm aboveground level. Host trapping during spring occurred from May 20th until May 30th, during summer from July 20th until July 30th, and during autumn from September 20th until September 30th of 2017 and 2018. Exceptionally, during the spring season of 2017, trapping occurred from June 1st until June 7th and only up to 700 m.a.s.l. due to large amounts of snow still present in both study areas. The traps were checked every 24 h, and activated traps were rebaited and reset. Live captures were euthanized by cervical dislocation. All captured individuals were stored in separately coded plastic bags and frozen at −20°C at the end of every collection day.

After each collection season, the animals were examined for ticks in the laboratory. The day before examination, hosts were removed from storage and left to thaw overnight in a 10°C cold room. The animals were then removed from the plastic bags and underwent a full‐body examination, starting with the head (snout, throat, neck, and ears), followed by the back and abdomen, legs, feet, and tail. Animals that were wet after thawing were first carefully dried using a hairdryer prior to the examination. The empty plastic bags were checked for ticks that might have dropped off the host while in storage. Attached or detached ticks were collected using tweezers and placed in a 1.5‐ml Eppendorf tube containing 70% ethanol solution. After examination, a lice comb was brushed through the fur against the hair orientation, and the animal was shaken by the tail above a white plastic tray for approximately 5 s in order to collect any ticks that might have been missed. Host species was determined using morphological and dental features (Van Der Kooij, 1999). The collected ticks were determined for life stage and species using a Zeiss Discovery V20 stereomicroscope, and using established reference keys (Arthur, 1963; Hillyard, 1996). After processing, each animal was resealed in newly coded bags and refrozen at −20°C for long‐term storage.

### 2.1 Data analysis

The statistical analysis of the data was performed in R version 4.0.2 (R Development Core Team, 2019). Because only a small number of *I*. *trianguliceps* adults (*n* = 78) and no *I*. *ricinus* adults were found on hosts, we only used larvae and nymphs in the analyses. As the residuals of the log ratios were centered around the mean, we used a mixed effects regression model with a normal distribution, using the propensity of ticks to parasitize either bank voles or common shrews as the response variable. We defined propensity (*P*) as the log ratio:

$$P=\log\frac{\left(\bar{{\mathrm{burden}}_{\mathrm{shrew}}}+0.05\right)}{\left(\bar{{\mathrm{burden}}_{\mathrm{vole}}}+0.05\right)}$$

where $\bar{{\mathrm{burden}}_{\mathrm{shrew}}}$ is the mean burden of ticks parasitizing common shrews$,\;\mathrm{and}\;\bar{{\mathrm{burden}}_{\mathrm{vole}}}$ is the mean burden of ticks parasitizing bank voles. Propensity would then be positive if mean burden of shrews was higher, negative if mean burden of voles was higher, and zero if both hosts have an equal mean burden. A propensity close to zero indicates that ticks parasitize hosts opportunistically, and a slope deviating from zero indicates a disproportional level of parasitism toward a certain host type (Figure 1). Because the function is undefined, if the mean burden of one or both hosts is zero, a small constant (0.05) was added to every estimated mean tick burden. The constant was chosen to limit the effect on the propensity when sample sizes are relatively small, and testing a wide range of constants (0.01–0.1) showed that 0.05 was statistically robust.

We pooled all captures from each capturing station (live and lethal traps combined), from each study area (Lifjell and Lærdal), and study year (2017 and 2018) in order to maximize the number of replicates in the analysis, using capturing station as the statistical unit in the analysis. Because each capture station was considered as a separate sample population, the maximum number of replicates in each dataset was 40 (2 years, 2 sites, and 10 stations), but no captures at a certain station or year resulted in fewer replicates in the datasets (number of replicates for *I*. *ricinus* larvae: *n* = 35; *I*. *ricinus* nymphs: *n* = 23, *I*. *trianguliceps* larvae: *n* = 33, and *I*. *trianguliceps* nymphs: *n* = 33). The main independent variables of interest were the proportion of bank voles in the entire host population (host population composition, consisting of voles and shrews), and the total host population size (the sum of all voles and shrews captured at that station per study area per year). We tested the propensity using four different datasets, one for each tick species and life stage (*I*. *ricinus* and *I*. *trianguliceps* larvae and nymphs). Year was used as a random factor, and in order to correct for any potential spatial dependency between the two study areas and each of the capturing stations, we used spatial autocorrelation in the model according to Zuur et al. (2009) with an exponential autocorrelation function from the R package *nlme* (Pinheiro et al., 2017). A *p*‐value smaller than .05 was considered significant.

We started with the null model, containing none of the predictor variables, then added host population composition and host population size individually, resulting in three competing models for each tick species and life stage. The AIC (Akaike information criterion) was then computed for each of the models. All models were fitted using a restricted maximum likelihood method, according to Zuur et al. (2009). Graphical output of the results was plotted using the R package *ggplot2* (Wickham, 2016).

## 3 RESULTS

**FIGURE 2:**
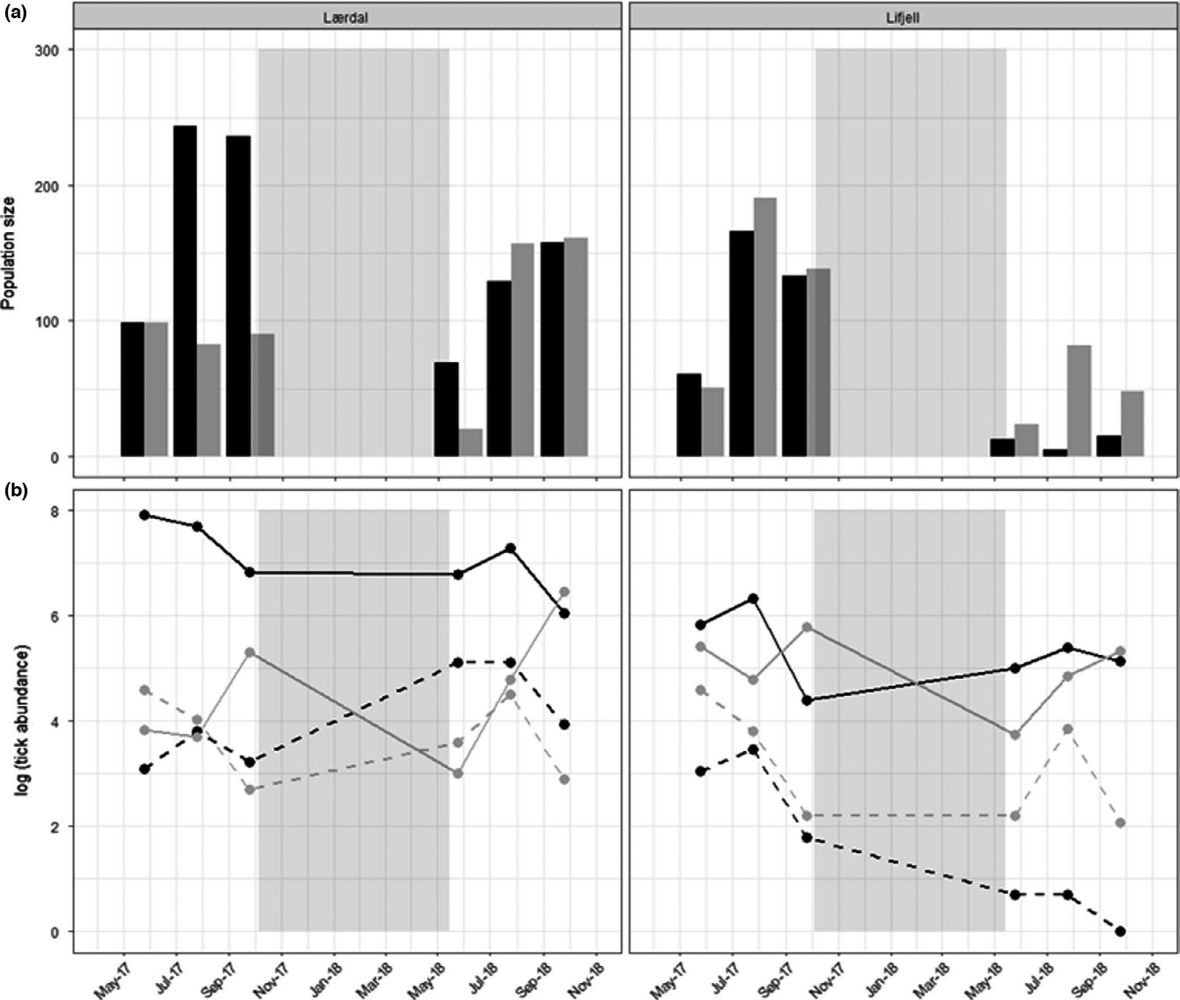
(a) Number of captures in each study area during the trapping periods (black bars: voles, gray bars: shrews), and (b) overall tick abundance (log) in each study area during the trapping periods (black lines: *I*. *ricinus*; gray lines: *I*. *trianguliceps*; solid lines: larvae; and striped lines: nymphs). The light gray rectangles represent the winter period (October 5, 2017 – May 15, 2018)

A total of 43,920 trap nights was performed during 2017 and 2018, capturing 2380 hosts (54.5% bank voles and 45.5% common shrews). A few other small mammal species were also captured, but these were not included in the analyses. A list of all species captured, and their respective burdens of both tick species is mentioned in De Pelsmaeker et al. (2021; Appendix 3). The captures in Lærdal were 1.7 times more numerous than in Lifjell (1544 and 927, respectively). From 2017 to 2018, bank vole captures dropped by 90.8%, and shrew captures by 59.5% in Lifjell, and in Lærdal bank vole captures dropped by 38.5%, whereas shrew captures increased by 23.9%. The reduced number of voles captured in both study areas indicate that the populations appear to be in a declining phase, and more so in Lifjell compared to Lærdal (Figure 2). A total number of 13326 ticks was collected (76.2% *I*. *ricinus* larvae, 4.1% *I*. *ricinus* nymphs, 15.8% *I*. *trianguliceps* larvae, and 4.0% *I*. *trianguliceps* nymphs), and ticks were 3.7 times more numerous in Lærdal compared to Lifjell (10,478 and 2848, respectively). The number of captured hosts and mean burden sizes for each study area and year are shown in Table 1.

**TABLE 1:** Number of bank voles and common shrews captured during 2017 and 2018 in both study areas, and mean burden size (±*SD*) on each host type per tick species and life stage

	Number of captures (%)		Total	*I.* *ricinus*				*I.* *trianguliceps*			
				Larvae		Nymphs		Larvae		Nymphs	
	*M. glareolus*	*S. araneus*		*M. glareolus*	*S. araneus*	*M. glareolus*	*S. araneus*	*M. glareolus*	*S. araneus*	*M. glareolus*	*S. araneus*
Lifjell											
2017	360 (48.6)	380 (51.4)	740	1.3 ± 3.9	1.3 ± 4.0	1.3 ± 0.6	0.0 ± 0.2	0.8 ± 2.4	1.0 ± 3.3	0.2 ± 0.5	0.3 ± 0.8
2018	33 (17.6)	154 (82.4)	187	3.0 ± 4.9	2.9 ± 6.8	0.0 ± 0.2	0.0 ± 0.2	1.8 ± 3.1	2.0 ± 4.7	0.6 ± 1.2	0.3 ± 0.9
Lærdal											
2017	579 (68.0)	272 (32.0)	851	5.7 ± 10.9	9.5 ± 18.5	1.1 ± 0.6	0.0 ± 0.2	0.2 ± 0.8	0.5 ± 2.2	0.2 ± 0.6	0.3 ± 0.9
2018	356 (51.4)	337 (48.6)	693	4.4 ± 12.2	3.5 ± 12.1	1.0 ± 3.0	0.1 ± 0.4	0.7 ± 1.9	1.6 ± 5.6	0.2 ± 0.7	0.2 ± 0.8

**FIGURE 3:**
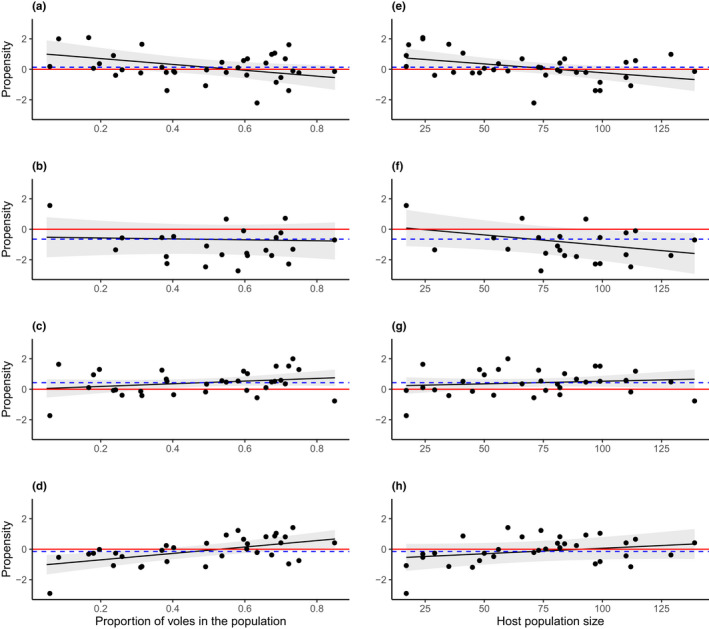
Model predictions showing the effect (restricted‐maximum likelihood) of the proportion of voles in the host population on the propensity of (a) *I*. *ricinus* larvae; (b) *I*. *ricinus* nymphs; (c) *I*. *trianguliceps* larvae; and (d) *I*. *trianguliceps* nymphs; and host population size on (e) *I*. *ricinus* larvae; (f) *I*. *ricinus* nymphs; (g) *I*. *trianguliceps* larvae; (h) *I*. *trianguliceps* nymphs parasitizing either voles or shrews. Positive values indicate a propensity toward shrews, negative values a propensity toward voles. Gray ribbons represent 95% confidence intervals. Blue dashed lines represent the null model intercept, red lines represent an equal propensity toward both host types. Dots represent the observed values

The model containing host population composition as a predictor variable best described the mean burden ratio (*P*) of *I*. *ricinus* larvae among voles and shrews (AIC = 96.40) (Table 2) and indicated that an increase in the proportion of voles in the host population favored the propensity toward voles (*t* = −2.156, *df* = 32, *p* = .039) (Table 3, Figure 3). Host population size also showed a significant result (*t* = −2.324, *df* = 32, *p* = .027), but the model fit was lower (AIC = 100.37) compared to that of the host population composition. The null model (representing the overall propensity), however, did not show any overall difference between host types (*t* = 0.589, *df* = 33, *p* = .560). In contrast to larvae, neither host population composition nor size had any significant effect on the propensity of nymphs to parasitize either host type (Table 3). Although the null model had the best fit to the observed data (AIC = 63.15), there was no difference in overall propensity toward voles or shrews either (*t* = −1.300, *df* = 21, *p* = .208).

**TABLE 2:** Summary of the Akaike information criterion (AIC) of the models used for each of the datasets. A 0 indicates absence in the model, and 1 indicates presence in the model. AICs in bold indicate lowest AIC

	Host population composition	Host population size	AIC
*I.* *ricinus* larvae	0	0	100.21
	1	0	**96.39**
	0	1	100.37
*I.* *ricinus* nymphs	0	0	**63.15**
	1	0	63.16
	0	1	69.75
*I.* *trianguliceps* larvae	0	0	88.09
	1	0	**87.28**
	0	1	98.52
*I.* *trianguliceps* nymphs	0	0	89.92
	1	0	**82.94**
	0	1	98.92

**TABLE 3:** Parameter estimates of the models assessing host propensity (*P*), indicating the mean burden ratio of ticks to parasitize shrews or voles, using a mixed‐effect model with a restricted maximum likelihood method

	Estimate	*SE*	*t*‐Value	*p*‐value
*I.* *ricinus* larvae				
Null model intercept	0.136	0.231	0.589	.560
Intercept	1.089	0.502	2.168	.038
Host population composition	−1.923	0.892	−2.156	.039
Intercept	0.932	0.394	2.364	.024
Host population size	−0.012	0.005	−2.324	.027
*I.* *ricinus* nymphs				
Null model intercept	−0.649	0.499	−1.300	.208
Intercept	−0.508	0.711	−0.715	.483
Host population composition	−0.307	1.048	−0.293	.772
Intercept	0.314	0.695	0.451	.657
Host population size	−0.014	0.007	−1.883	.074
*I.* *trianguliceps* larvae				
Null model intercept	0.429	0.139	3.076	.004
Intercept	0.009	0.335	0.026	.980
Host population composition	0.875	0.637	1.373	.180
Intercept	0.182	0.342	0.532	.598
Host population size	0.003	0.004	0.790	.436
*I.* *trianguliceps* nymphs				
Null model intercept	−0.146	0.441	−0.331	.743
Intercept	−1.119	0.353	−3.168	.004
Host population composition	2.106	0.626	3.363	.002
Intercept	−0.658	0.520	−1.266	.215
Host population size	0.007	0.005	1.315	.198

Although *I*. *trianguliceps* larvae showed an overall propensity to parasitize voles less than shrews, as indicated by the null model (*t* = 3.076, *df* = 31, *p* = .004), the AIC was slightly higher than that of the model containing host population composition (88.09 and 87.28, respectively). However, neither the composition nor size of the host population showed a significant effect on the propensity of parasitism toward voles or shrews (Table 3). Similarly, neither the null model nor the host population size showed any effect on the propensity of *I*. *trianguliceps* nymphs (Table 3), but the model containing the host population composition had the best fit to the data (AIC = 82.94) and showed that an increase in the proportion of voles corresponded to a significant increase in propensity toward shrews (*t* = 3.363, *df* = 30, *p* = .002) (Figure 3).

## 4 DISCUSSION

In this study, we studied common shrews as a potential alternative small mammal host to assess whether ticks would have the propensity to parasitize shrews equally or more if vole populations were at low densities. We hypothesized that opportunistic ticks would have an equal likelihood of parasitizing a passing host, regardless of the host species, and therefore no pattern would be observed in the mean burden ratio (propensity) as the host population changes in size and host composition. In both study areas, the number of captured voles dropped substantially from one year to the next, while shrew captures dropped less, or even increased. This could indicate that the shrew populations fluctuate less than voles, as would be expected from previous studies (Buckner, 1969; Henttonen et al., 1989), and that in both areas, the vole populations appear to be in a declining phase of the population cycle.

Although the proportion of voles in the population as well as the overall host population size were considered as model predictors, the total host population size was not a good predictor of propensity. Furthermore, as the host population size appears to be mainly dependent on the fluctuation of voles, the proportion of voles in a population and the overall host availability seems to be somewhat correlated.

*Ixodes ricinus* larvae showed a significant propensity toward voles as their proportion increased in the host population, indicating that these ticks parasitize voles disproportionally more often when voles are common, but that when vole populations are on the decline, these larvae utilize alternative hosts such as shrews at higher rates, thus maintaining their capacity to establish and disperse. Similar to all life stages of the nidicolous *I*. *trianguliceps*, *I*. *ricinus* larvae do not disperse far from the place where the egg batch hatched, and groups of larvae are often aggregated in the landscape (Nilsson & Lundqvist, 1978). A larger abundance of a specific host type could therefore increase the likelihood of encounter with questing larvae, but would likely also result in a higher propensity on that host type. The higher levels of parasitism on voles at high vole proportions could demonstrate the potential of *I*. *ricinus* larvae to display a functional response in which the most common host becomes disproportionately more parasitized.

The proportion of voles in a population appeared to have no effect on the propensity of *I*. *ricinus* nymphs to parasitize either host type, and the null model indicated no difference in propensity toward either host type. The absence of a change in propensity toward either host type as the rodent density changes indicates that shrews are suitable hosts, enabling the maintenance and further spread of ticks, similar to larvae. However, we had no information on the availability of other mammalian or avian hosts, and the generalist *I*. *ricinus* is able to parasitize species other than small mammals (Talleklint & Jaenson, 1997). A change in the abundance of such other species may also lead to an increased utilization of these hosts by nymphs. Although *I*. *ricinus* larvae have a tendency to parasitize small mammals, they are also found feeding on large ungulates such as, for example, cervids (Talleklint & Jaenson, 1998) and birds (Humair et al., 1993; Marsot et al., 2012). This generalist behavior allows ticks of any life stage to feed on a variety of hosts, and may enable them to progress further northwards despite the population fluctuations of small mammal hosts. It may also allow ticks to progress further in altitude, where the abundance of large mammals might be reduced (Estrada‐Peña & de la Fuente, 2017; Gilbert, 2009). This generalist ability may have contributed to *I*. *ricinus* becoming the most widespread tick species in Europe and may enable it to quickly spread to new areas with increased global warming.

In the case of *I*. *trianguliceps* larvae, the proportion of voles in the host population had no effect on the propensity to parasitize either host type, and the null model showed that voles were overall significantly less parasitized than shrews. Although generally considered an endophilic tick species, *I*. *trianguliceps* larvae have been reported to be somewhat exophilic, questing for hosts in harborages and animal trails (Hillyard, 1996). Studies have yet to confirm whether any stage of *I*. *trianguliceps* is indeed exophilic; as to the best of our knowledge, no *I*. *trianguliceps* ticks have been collected through the flag dragging or flagging method (Randolph, 1975b). However, these results may also indicate that larvae could behave opportunistically, similar to *I*. *ricinus* larvae. Our data suggest that *I*. *trianguliceps* larvae can parasitize both hosts, irrespective of their density, but seem to parasitize shrews more commonly overall. The potential for larvae of both tick species to utilize shrews as host species during the low phases in rodent population cycles may also facilitate the possibility to acquire tick‐borne pathogens during this life stage from reservoir hosts.

A change in the proportion of available voles did significantly influence the propensity of *I*. *trianguliceps* nymphs. Paradoxically, nymphs appeared to be more commonly found on the least abundant host, the shrew, when the proportion of voles in the host population was higher. *I*. *trianguliceps* is a strict small mammal specialist with an endophilic behavior, parasitizing small mammals during all life stages (Hillyard, 1996; Randolph, 1975a) residing inside host burrows and attaching or dropping off hosts inside the burrows (Bown et al., 2006). During declines in rodent populations, rodent burrows may become vacant for shrews to occupy, as shrews have been shown to make extensive use of rodent burrows (Crowcroft, 1955). As shrew populations fluctuate less than rodents, they may occupy some burrows left vacant after a rodent peak, as shrews have been found to forage in rodent burrows and runways (Gliwicz & Taylor, 2002). *I*. *trianguliceps* nymphs in burrows formerly occupied by voles and now by shrews may then parasitize available shrews, but burrows left vacant may leave nymphs without a host to feed on. Depending on climatic conditions, the life cycle of *I*. *trianguliceps* is typically 2 to 5 years (Balashov, 1997), and the absence of a host could cause a larvae or nymph to deplete its energy reserves in less time. As ticks are dependent on host movement for dispersal (Medlock et al., 2013), it may take a certain amount of time for voles to recruit *I*. *trianguliceps* and allow for ticks to colonize new or existing burrows, where they may previously have gone extinct due to a prolonged period of host absence. In some predator–prey systems, when a prey species becomes more abundant, the predator may switch to the least abundant prey if the disadvantages of predating the more abundant species become too large (Tallian et al., 2017). Bank voles have been found to develop an acquired resistance against tick infestation (Dizij & Kurtenbach, 1995), and perhaps a similar mechanism may cause *I*. *trianguliceps* nymphs to select voles disproportionally less as vole numbers increase and develop an immune response against tick infestation.

Peaks and low points in bank vole population cycles in Norway are usually 3–4 years apart (Myrberget, 1973), but in recent years these cycles seem to have become more erratic (Hörnfeldt et al., 2005). Warming temperatures may reduce or disrupt the amplitude and frequency of rodent cycles, reducing the fluctuations of rodent hosts. A reduction in amplitude could entail that low points in the cycles become less extreme, and overall more hosts are available for ticks to feed on. Smaller‐amplitude cycles could therefore facilitate the persistence of ticks, particularly at higher altitudes. Although vole densities in both study areas substantially decreased from 2017 to 2018, indicating a declining phase of the cycle, we cannot say how much further the populations would decline. In addition, the number of available rodents at different stages of the population cycle may affect the infestation rates between the two host species. One would, for example, expect that when rodent populations reach a low point in the cycle, the propensity toward shrews would be more pronounced as shrews will then be the most common host available. To get a clearer understanding on how these host dynamics might influence the survival and dispersal of ticks in northern regions, studies encompassing two or more complete rodent cycles are needed.

A large amount of tick burden data were used to create the different datasets in this study, and we believe that the results reliably reflect the host–parasite dynamics, although with some notable limitations. For example, as not all tick larvae successfully feed on a host and molt to the next life stage, and nymphs are generally less abundant than larvae, the sample sizes for nymphs of both tick species in this study are smaller in comparison to larvae. In addition, *I*. *ricinus* is considered a generalist parasite, and nymphs parasitize hosts other than small mammals such as intermediate size mammals, as well as large wild ungulates (Medlock et al., 2013; Talleklint & Jaenson, 1997). This may have further limited the number of *I*. *ricinus* nymphs in the study, and since we focused solely on tick burdens found on small mammals, the *I*. *ricinus* nymphal burdens may not be representative of the full cohort of ticks in the environment. Thus, it is possible that a larger sample size of nymphs may have resulted in slightly different results. Burden data on other host species sampled in the same area may indicate if the propensity of nymphs shifts to another small mammal, or to other host species that were unaccounted for in this study. Also, the numbers of captured animals may not accurately reflect the actual population size or host proportions in the focal study areas (Kikkawa, 1964). As both live and lethal traps were baited for voles and not for shrews, this may have introduced a bias in the estimations of the hosts available to ticks. Additional baiting for shrews (e.g., meat or cat food) may increase the number of shrews captured and affect population estimates. Conversely, additional bait may also cause rodents to behave differently toward the traps, affecting rodent captures (Taylor et al., 1974). Animals may also grow beyond the trap capacity, therefore only a certain cohort of the population would be susceptible to capture (Leslie et al., 1953). However, in this study, the trap capacity of both trap types was large enough to allow for the capture of bank voles and common shrews of any size. By collapsing the samples to station level over all three trapping seasons, we encompassed seasonal variation in home ranges. Additionally, as animals were only trapped once and removed from the population, no trap shyness was induced. Although there may be some differences between the number of captures and the actual population size and host proportions, we believe that our capture rates are more or less representative of the actual host population.

## 5 CONCLUSION

In areas characterized by high‐amplitude rodent cycles, shrews may provide a supplementary source of hosts, allowing ticks to persist at higher altitudes as climatic conditions become more favorable. As the effects of climate change are expected to be exacerbated in northern regions (Houghton, 1996), the relative stability of shrew populations may allow ticks to expand toward new areas in Norway, despite large periodic differences in rodent availability. However, the dynamics of host availability and tick persistence are complex, and the effects of a changing climate on rodent cycles may change the propensities at which ticks parasitize hosts. The changing propensity of *I*. *ricinus* larvae to parasitize different hosts as their proportions change demonstrates the potential of shrews to serve as reserve hosts for ticks if rodent populations decline, and for shrews or other alternative hosts to maintain tick populations. As *I*. *trianguliceps* feeds exclusively on small mammals, shrews may play an even more important role in the maintenance of this species when rodent availability declines, and larvae may have the same potential as *I*. *ricinus* in utilizing other hosts. As climate change may not only facilitate tick range expansion but may also affect the amplitude and frequency of rodent cycles, the presented results may act as a starting point for further long‐term observations. Further investigation of parasite–host relations in regards to host population dynamics and the effects of climate change thereon may provide valuable insights in the northward and upward tick progression and the accompanying disease risks.

## AUTHOR CONTRIBUTIONS

**Nicolas De Pelsmaeker:** Formal analysis (equal); Writing – original draft (equal). **Lars Korslund:** Conceptualization (equal); Formal analysis (equal); Supervision (equal); Validation (equal); Writing – review & editing (equal). **Øyvind Steifetten:** Conceptualization (equal); Project administration (equal); Supervision (equal); Writing – review & editing (equal).

## Data Availability

Datasets used in the analysis: FigShare https://doi.org/10.23642/usn.16836655.
